# Une forme pseudo-tumorale du neuro-Behçet: à propos d'un cas et revue de la littérature

**DOI:** 10.11604/pamj.2019.33.194.18719

**Published:** 2019-07-12

**Authors:** Mohammed Guini, Mohammed Khoulali, Nabil Raouzi, Noureddine Ouali, Fayçal Moufid

**Affiliations:** 1 Service de Neurochirurgie, CHU Mohammed VI Oujda, Oujda, Maroc

**Keywords:** Behçet, manifestation neurologique, tumeur cérébrale, Behçet, neurological manifestation, brain tumor

## Abstract

Pas toute masse intra cérébrale n'est nécessairement une tumeur. Nous décrivons un cas de patient suivi pour maladie de Behçet qui se présente avec un tableau de processus expansif intracrânien révélé par une hémiparésie droite avec des signes d'hypertension intracrânienne; l'imagerie par résonance magnétique (IRM) cérébrale a objectivé une lésion faisant penser à une tumeur gliale, mais les contextes cliniques, biologiques et radiologiques ont permis d'établir le diagnostic de forme pseudo tumorale de neuro-Behçet. Cette description est suivie d'une analyse clinique à la lumière de la littérature disponible sur la pathologie.

## Abstract (en)

Mass lesions of the brain are not always a tumor. We report the case of a patient followed for Behcet syndrome presenting with expansive intracranial process revealed by right hemiparesis associated with signs of intracranial hypertension. Brain magnetic resonance imaging (MRI) showed a lesion suggesting glial tumor. Clinical and radiological assessments as well as laboratory tests helped to establish the diagnosis of pseudo-tumor form of neuro-Behçet disease. This study report is followed by a clinical analysis based on the available literature on this disorder.

## Introduction

La maladie de Behçet est une vascularite systémique rare, d'étiologie inconnue décrite pour la première fois en 1937, avec prédominance de lésions cutanéo-muqueuses et oculaires [1]. Les manifestations neurologiques observées chez 1 à 59% des cas selon les séries constituent le syndrome de neuro Behçet avec des formes “parenchymateuses” et “extra-parenchymateuses” [2]. L'expression pseudo-tumorale est rare voire exceptionnelle, rapportée dans la littérature comme des cas isolés, posant un problème de diagnostique différentiel avec les tumeurs cérébrales [3].

## Patient et observation

Il s'agit d'un patient âgé de 48 ans, avec antécédent de cardiopathie rhumatismale compliquée d'une valvulopathie mitrale, suivi depuis 22 ans pour Behçet cutané sous colchicine avec notion d'aphtose bipolaire récidivante, qui présente depuis 03 mois une lourdeur de l'hémicorps droit avec aphasie d'évolution progressive associée à des signes d'hypertension intracrânienne fait de céphalées, vomissements, évoluant dans un contexte de conservation de l'état général.

**Figure 1:**
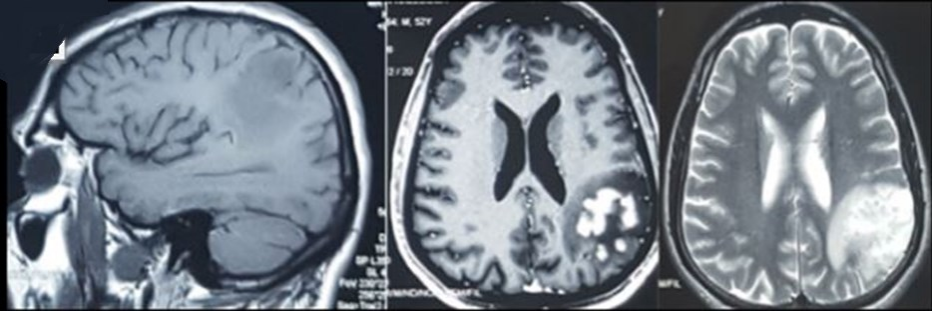
IRM cérébrale initiale (T1, T1 Gado, T2): une lésion pariétale postérieure gauche hypointense en T1, hyperintense en T2, flair et en diffusion en doigt de gant cortico sous corticale prenant le contraste sous forme nodulaire

L'examen clinique a objectivé un patient conscient aphasique avec une hémiparésie droite, et des aphtoses génitales avec des nodules sous cutanées au niveau du membre inférieur droit en rapport avec un lipome. Le bilan ophtalmologique était sans anomalie ne montrant pas d'uvéite avec un fond dœil normal. Le bilan biologique standard était sans anomalie, avec liquide céphalo-rachidien (LCR) normal. L'imagerie par résonance magnétique (IRM) cérébrale a montré une lésion pariétale postérieure gauche hypointense en T1, hyperintense en T2, flair et en diffusion en doigt de gant cortico sous corticale prenant le contraste sous forme nodulaire (Figure 1). L'aspect radiologique a évoqué en premier une tumeur gliale de bas grade (mais le diagnostic a été redressé vu le contexte clinique), l'antécédent de Behçet cutané et l'existence d'aphtose bipolaire. Le traitement initial était à base de bolus de méthylprednisolone avec surveillance clinique et radiologique.

**Figure 2:**
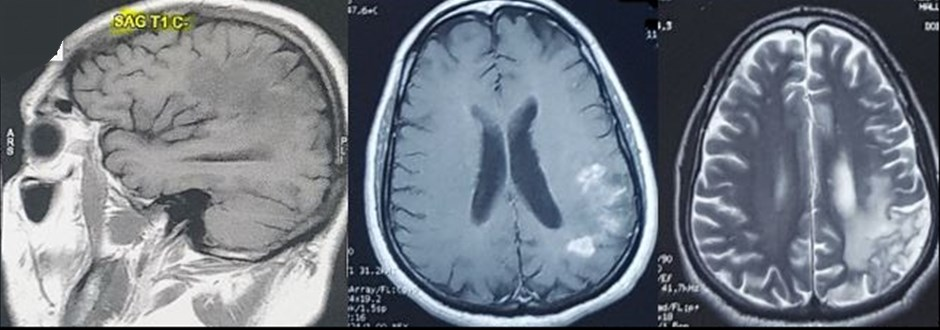
IRM cérébrale de controle (T1, T1 Gado, T2): des nodules multiples actifs pariétale gauche avec oedème péri lésionnel en rapport avec un neuro-Behçet évolutive

**Figure 3:**
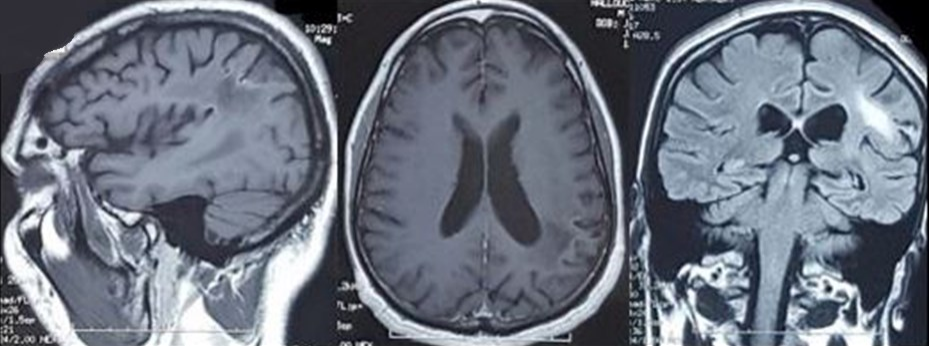
IRM cérébrale (T1, T1 Gado, T2 Flair): régression des lésions

**Evolution:** après trois bolus de corticothérapie forte dose, on a noté une amélioration sur le plan clinque avec récupération totale du déficit neurologique et de l'aphasie. L'IRM de control a objectivé des nodules multiples actifs pariétaux gauches avec œdème péri lésionnel en rapport avec un neuro-Behçet évolutif (Figure 2). Un traitement par bolus de cyclophosphamide a été instauré (dose d'induction 200 mg/j pendant 10 jours puis 1 g/bolus mensuel pendant 06 mois) avec un control radiologique montrant un nettoyage de la lésion (Figure 3).

## Discussion

**Présentation clinique:** le syndrome neuro-Behçet selon l'International Consensus Recommandations (ICR) [4] est l'ensemble des atteintes neurologiques survenant chez un patient ayant souffert ou souffrant de tous les autres symptômes systémiques de la maladie de Behçet. Cette entité retrouvée chez 5,3 à plus de 50% des patients selon les séries regroupant les «atteintes parenchymateuse» et «atteintes extra-parenchymateuses». La forme pseudo-tumorale du neuro-Behçet a longtemps été rapportée au sein de cas isolés. À ce jour, 34 cas de forme pseudo-tumorale ont été rapporté dans la littérature scientifique. Les Tableau 1 et Tableau 1 (suite) résument les caractéristiques cliniques radiologiques et thérapeutiques de ces lésions. D'après Noel N *et al.* [5], cette atteinte comptait pour 1,8% des cas de neuro-Behçet, avec une prédominance masculine et avait des caractéristiques cliniques propres: les signes pyramidaux et l'hémiparésie semblent plus fréquents que dans les formes parenchymateuses classiques. Elle peut être inaugurale chez un tiers des cas, posant ainsi un réel problème de diagnostic différentiel avec une tumeur cérébrale.

**Tableau 1:** Caractéristiques cliniques des cas rapportés dans la littérature

Auteur	Age / sexe	Délai (ans)	Localisation	Ponction lombaire	Anatomo-pathologie	Traitement	Résultats
**Litvan 1987**	51 M	30	Pariéto-occipitale gauche	Hyperprotéinorachie Cuture négative	-	Glucocorticoïdes	Amélioration partielle
**Neudorfer 1993**	27 M	-	Noyau lenticulaire droit	-	-	Glucocorticoïdes	Amélioration
**Geny 1993**	-	-	Capsulo-thalamique	-	Non spécifique	Glucocorticoïdes	Amélioration
**Visaga 1996**	16 F	2	Jonction bulbo médullaire/Pédoncules cérébraux	Non	Non	Glucocorticoïdes, chlorambucil	Amélioration
**Yoshimura 2001**	41 F		Région thalamo-lenticulaire gauche	Pléocytose	Non spécifique	Glucocorticoïdes	Amélioration
**Ben Taarit 2002**	26 F	-	Protubérance /Pédoncule cérébral droit	-	Non	Glucocorticoïdes	Amélioration
**Imoto 2002**	50 M	-	Ganglions de la base, Tronc cérébral et la substance blanche	Non	Infiltration péri vasculaire Cellules inflammatoires	3 bolus de corticoïdes	Réduction de la taille
**Park 2002**	52 F	-	Hémisphère Cérébelleux droit s'étendant au 3ème ventricule	Lymphocytes 25 / L, Protéines 300 mg / dl, Glucose normal	Vascularite lymphocytaire	Glucocorticoïdes, azathioprine	Amélioration initiale. Trois épisodes de récurrence. Décès
**Tuzgen 2003**	59 F 45 F	- 03 mois	Fronto-temporale droite Jonction diencéphalo-mésencéphalique gauche	Non Non	Gliose, vascularite, Thrombose Prolifération vasculaire Non	Exérèse chirurgicale Glucocorticoïdes	Amélioration radiologique Hémiparésie persistante Amélioration
**Bennett 2004**	23 M	-	Lobe temporal gauche, Pédoncule envahissant, thalamus interne capsule, ganglions de la base	Non	Infiltration inflammatoire péri vasculaire	Glucocorticoïdes, azathioprine	Amélioration clinique et radiologique
**Matsuo 2005**	33 M	7 ans	Ganglions de la base	Pléocytose 26 mm3 Glucose normal protéines normal	Gliose Infiltrations inflammatoires	03 bolus de corticoïdes	Amélioration
**Schmolck 2005**	39 M	5 ans	Thalamus gauche	Non	Non	Dexaméthasone, Bolus mensuel de cyclophosphamide	Amélioration radiologique
**Darmoul 2006**	38 M	-	Thalamus gauche	-	Non	Glucocorticoïdes Immunosuppresseur	Amélioration radiologique
**Appenzeller 2006**	43 F	20 ans	Thalamus droit Noyaux lenticulaire	Non	Gliose avec astrocyte gemistocytique	Dexaméthasone méthylprednisolone prednisone oral	Amélioration
**Kösters 2006**	30 M	-	Fronto pariétale	LCR normal	Vascularite lymphocytaire des petits vaisseaux	Dexaméthasone, azathioprine	Amélioration réduction de la taille de la lésion

**Tableau 1 (suite):** Caractéristiques cliniques des cas rapportés dans la littérature

Auteur	Age / sexe	Délai (ans)	Localisation	Ponction lombaire	Anatomo-pathologie	Traitement	Résultats
**Heo 2008**	47 M	10 ans	Masses multiples de la région pontique gauche, Cortex pariétal	Non	Infiltrat périvasculaire Lymphocytaire,	Glucocorticoïdes fortes doses	Réduction de la taille de la lésion
**Varoglu 2010**	38 M	-	Le mésencéphale, Ganglions de la base bilatéraux, Bras postérieur de la capsule interne	Non	Pas de cellules tumorales	-	Amélioration
**Bouomrani2010**	45 M	17 ans	Tronc cérébral		Non	Cyclosporine, prednisone, cyclophosphamide	Amélioration
**Shapiro 2012**	30 M 30 M	03 ans 10 ans	Capsulo-lenticulaire Pédoncules	Méningite Aseptique Pléocytose		Prednisone, cyclophosphamide Glucocorticoïdes, infliximab	Bons résultats
**Martínez-Estupinan ˜ 2014**	23 M	04 ans	Cervelet	Méningite Aseptique	Changements de réactifs	Glucocorticoïdes, azathioprine, adalimumab, subsequent cyclophosphamide, rituximab	Réponse faible
**NAZIF 2014**	27 F	08ans	Pons			Cyclophosphamide Prednisone	Régression complète de la lésion
**SERKAN 2015**	27 F		Thalamus Gauche	LCR normal		Méthylprednisolone Azathioprine	Amélioration partielle
**J. Jade 2016**	38 F	06 ans	Plexus choroïde Corne temporale du ventricule latéral droit	Méningite lymphocytaire Culture négative	Infiltration inflammatoire péri vasculaire	Cyclophosphamide, prednisone Rituximab	Amélioration clinique et radiologique

**Imagerie:** classiquement selon Leclercq D *et al.* [6], les lésions cérébrales de neuro-Behçet apparaissent en IRM, comme des zones hypo à iso intense en séquences T1, avec un hypersignal en séquence pondérée T2, de taille variable, parfois confluentes et étendues, touchant préférentiellement les noyaux de la base, le mésencéphale et le diencéphale, avec une extension possible les capsules internes. Les lésions récentes peuvent présenter un effet de masse, une augmentation du contraste et par conséquent cela donne un aspect pseudo-tumoral [7]. Les principaux diagnostics différentiels sont les tumeurs gliales, les lymphomes ou les lésions infectieuses et granulomateuses.

**Intérêt de la biopsie:** certains auteurs recommandent la biopsie stéréotaxique pour écarter une tumeur cérébrale, mais elle n'est pas nécessaire pour le diagnostic de la maladie de Behçet d'autant plus si le contexte clinique est évocateur de celle-ci [8]. Dans la forme inaugurale et en absence de signes classique de la maladie, la biopsie peut être discutée et qui peut montrer des lésions souvent non spécifiques et incluent des infiltrats périvasculaires de lymphocytes et d'histiocytes CD 68 (+) [9].

**Traitement:** le traitement de référence de la maladie de neuro-Behçet est la méthylprednisolone administrée à forte dose pendant 7 à 10 jours, suivi d'une diminution progressive des doses orales sur une période de 3 à 6 mois, en fonction de la gravité de la rechute. Ce traitement a donné de bons résultats dans les lésions du tronc cérébral et dans la forme parenchymateuse. Le traitement par les anti-inflammatoires à long terme a été administré avec des agents immunosuppresseurs traditionnels, tels que l'azathioprine, la salazopyrine et d'autres dérivés de l'acide 5-aminosalicylique [10], ainsi qu'avec la cyclosporine. La thérapie biologique et la thérapie par anticorps monoclonal ont fait l'objet de nombreuses études; parmi ces traitements, les inhibiteurs du facteur de nécrose tumorale α tels que l'infliximab, l'étanercept et l'adalimumab ont montré des effets bénéfiques [11].

## Conclusion

La forme pseudo tumorale de neuro-Behçet est rare, qui peut survenir lors de l'évolution de la maladie orientant ainsi le diagnostic ou inhabituellement d'une façon inaugurale. Une biopsie peut être envisagée pour éliminer une tumeur cérébrale en l'absence de signes cliniques évocateurs de la maladie de Behçet.

## Conflits d’intérêts

Les auteurs ne déclarent aucun conflit d'intérêts.
